# The Impact of COVID-19 on Lombardy Region ST-Elevation Myocardial Infarction Emergency Medical System Network—A Three-Year Study

**DOI:** 10.3390/jcm11195718

**Published:** 2022-09-27

**Authors:** Giuseppe Stirparo, Lorenzo Bellini, Giuseppe Ristagno, Rodolfo Bonora, Andrea Pagliosa, Maurizio Migliari, Aida Andreassi, Carlo Signorelli, Giuseppe M. Sechi, Nazzareno Fagoni

**Affiliations:** 1 Faculty of Medicine, School of Public Health, University of Vita-Salute San Raffaele, Via Olgettina 60, 20090 Milano, Italy; 2 Agenzia Regionale Emergenza Urgenza Headquarters (AREU HQ), Via Campanini 6, 20090 Milano, Italy; 3 Dipartimento di Fisiopatologia Medico-Chirurgica e dei Trapianti Via Festa del Perdono 7, 20122 Milano, Italy; 4 Fondazione IRCCS Ca’ Granda Ospedale Maggiore Policlinico, 20090 Milano, Italy; 5 Dipartimento di Medicina Molecolare e Traslazionale, Università di Brescia, Piazza del Mercato, 15, 25121 Brescia, Italy

**Keywords:** emergency care, COVID-19, STEMI, Lombardy region

## Abstract

*Objectives*: The COVID-19 pandemic had a significant impact on emergency medical systems (EMS). Regarding the ST-elevation myocardial infarction (STEMI) dependent time network, however, there is little evidence linked to the post-pandemic phase regarding this issue. Such information could prove to be of pivotal importance regarding STEMI clinical management, especially pre-hospital clinical protocols such as fibrinolysis. *Methods*: A retrospective observational cohort study of all STEMI rescues recorded in the Lombardy EMS registry from the 1st of January 2019 to the 30th of December 2021. *Results*: Regarding the number of STEMI diagnoses, March 2020 (first pandemic wave in Italy) saw a reduction compared to March 2019 (OR 0.76 [0.60–0.93], *p* = 0.011). The average time of the entire mission increased to 63.1 min in 2021, reaching 64.7 min in 2020, compared with 57.7 min in 2019. The number of HUBs for STEMI patients saw a reduction, falling from 52 HUBs in the pre-pandemic phase to 13 HUBs during the first wave. *Conclusions*: During the pandemic phase, there was an increase in the transportation times of STEMI patients from home to the hospital. Such changes did not alter the clinical approach in the out-of-hospital phase. Indeed, the implementation of fibrinolysis was not required.

## 1. Introduction

Italy was one of the first European countries affected by the COVID-19 pandemic [1], which resulted in a government-imposed general lockdown [2].

Lombardy, the most populous region in Italy with about 10 million inhabitants, was the first area involved in the outbreak [3,4], followed by other areas of Europe [5,6].

Following the COVID-19 pandemic, the Lombardy Emergency Medical System (EMS), managed by the Agency for Emergencies (AREU), underwent profound changes compared to the previous, pre-pandemic, period [7,8,9]. One of the main issues was the higher number of requests for intervention, which increased by 51% [9] and a significant change in the HUB and Spoke network, due to the necessity to ensure hospitals dedicated only to patients suffering from COVID-19 and others to patients with time-dependent diseases [10,11]. In addition, a great variability was observed among the countries affected by COVID-19 in the number of time-dependent pathologies, i.e., myocardial infarction [12,13], stroke [14,15] and major trauma [16], with an overall decrease being noted. At the same time, cardio-circulatory arrest [17] and respiratory failure increased [9,18].

In time-dependent disease, the EMS plays a central role, with the time of transport and the clinical treatment being two important aspects for patient outcome. In ST-elevation myocardial infarction (STEMI), according to general guidelines, the time between the first medical contact and the percutaneous transluminal coronary angioplasty (PTCA) should be less than 120 min; if the transportation time happens to be longer, fibrinolysis is then indicated. Thus, EMS intervention time plays a central role in STEMI, up to the point of modifying the therapeutic priority [19,20,21,22].

During the outbreak, a stark reduction in STEMI diagnoses in Emergency Departments (ED) was reported all over Europe, probably related to the stay-at-home policy [23,24,25,26,27] and the reported increase in overall duration of EMS rescue missions [28,29], to such an extent that the introduction of fibrinolysis was hypothesized [30,31]. An increase in deaths related to STEMI was also reported [32] in many studies and this phenomenon was linked to an increase in mortality for out of hospital cardiac arrest [33,34] and other major changes [35,36].

Nevertheless, data regarding the post-pandemic phase is lacking.

The aim of this study is to analyze the impact of COVID-19 on STEMI management by EMS in the Lombardy region compared to the pre-pandemic period. The analysis aims to prove whether the EMS efficiency standards were equal to the pre-pandemic phase.

## 2. Methods

This is a cross-sectional study. The study was conducted in accordance with the principles of the Helsinki declaration and was approved by the AREU Data Protection Officer, in January 2022.

### 2.1. Data Registry

Data were provided by the Lombardy AREU headquarters registry. The data analysis process was conducted employing the SAS-AREU portal.

For every patient that calls the EMS system in the Lombardy region, the technician who answers the call activates a mission-card in the SAS-AREU portal. Demographic data are manually entered into a mission-card, and the telephone number and location are identified with the telephone’s GPS method. The technician can send a BMV or MSA based on the health problem. The vehicle trip is recorded on SAS-AREU and linked to the patient’s mission-card. For correct identification of the patient, the BMV or MSA crews, after a clinical examination, call the Doctor or nurse in medical’s central for defined the hospital destination. The trip time and the location of the vehicle are recorded on the mission-card. At the of mission al medical or nurse in medical’s central complete the mission-card with all demographic characteristics.

All data, about the trip or clinical examination are recorded in the SAS-AREU portal and are linked in one mission-card connected to the demographic characteristics of the patient.

The portal contains all data regarding emergency calls, and the scenarios involving STEMI were selected.

According to AREU internal guidelines, an ECG is performed by the BMV crew on all patients with chest pain. All ECGs are sent to the doctor present in SOREU, for validation. If the ECG is positive for STEMI, it is also sent to the cardiology operating unit where the patient will be then transported immediately by BMV.

We analyzed three different variables, the first was the number of annual diagnoses, defined as the number of patients with a positive ECG for STEMI. The second was average time of the EMS first vehicle on the scene, defined as the time elapsed from the time the vehicle left the station to the time it arrived at the patient’s site.

The last one was average time of duration of mission, considered as the time between the start of the mission, in which the vehicle left the station, and arrived at the destination hospital.

Study period from 1 January 2019 to 30 December 2021.

### 2.2. EMS STEMI System

In the Lombardy region, to be able to recognize STEMI patients quickly, all the Basic Life Support Medical Vehicles (BMV), the first tier of AREU EMS, have been equipped with electrocardiographs to acquire and forward a 12-leads ECG. According to AREU’s instructions, all BMV crews must perform an ECG in patients presenting with chest pain. They also must do it whenever requested by a doctor in the SOREU station.

The doctor in the SOREU station receives the ECG via a telemedicine system. If a STEMI is identified, the physician decides the patient’s destination and chooses the closest HUB to the rescue site from STEMI Network [36].

The transport time is recorded from the location of the BMV and is collected by the GPS system automatically.

The head of the BMV’s crew must mark the vehicle status (i.e., free for other patients, with the patient on board, in hospital) to proceed in the EMS mission. The status is necessary for the patient’s rescue decision, and it is registered in the AREU system, with the time of the mission, to be used to calculate the overage time of the mission and the time of the first vehicle on scene.

The position of the BMV is defined through the GPS system.

All data are collected automatically by the AREU data collection system.

### 2.3. Statistical Analysis

The categorical variables are presented as number, and the continuous variables are presented as mean and standard deviation (SD). The categorical variables were analyzed by means of χ^2^ test, and the relative odds ratios (OR) and 95% confidence intervals (CI95%) were provided. Continuous variables were tested for normality by means of the Kolmogorov–Smirnov test and *t* test for independent data was applied to compare the average time of first vehicle on scene in 2020 and 2021 to 2019, and to compare the average time duration of the missions in 2020 and 2021 to 2019. ANOVA test was applied to evaluate the average number of STEMI diagnoses per month.

Differences were considered significant with *p* < 0.05, otherwise, they were considered non-significant (NS). The Prism 8.0.1 statistical software (GraphPad Software LLC, San Diego, CA, USA) was used to this aim.

## 3. Results

In 2019, the EMS transported 2174 patients with STEMI to the closest hospital provided with a cath lab; 1774 (81%) were male with an average age of 66.1 ± 13.4 years. In 2020, 2156 STEMI patients were rescued, 1599 were male (75%), with an average age of 65.5 ± 12.9 years. In 2021, 2238 STEMI patients,1821 male (81%) (average age 64.7 ± 12.7 years) were transported. (NS compared to 2019 and 2020).

The average number of STEMI diagnoses per month was not different between the pre-pandemic and the pandemic period, in the three years of study (181 vs. 179 vs. 186, *p* = 0.74).

**Figure 1:**
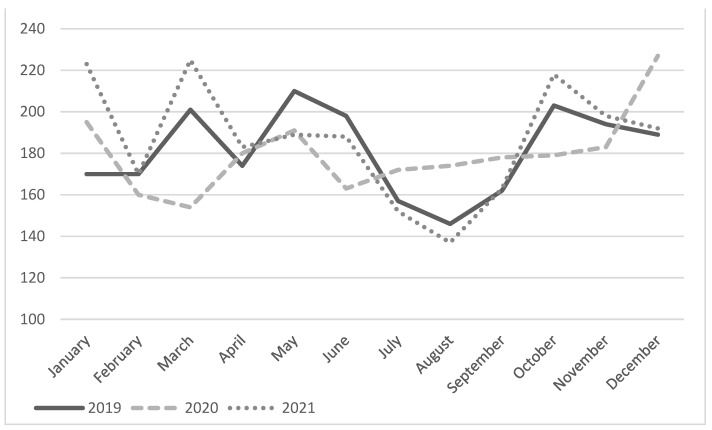
Monthly diagnoses of STEMI during the three years analyzed.

Figure 1 shows the number of STEMI diagnoses per month in the reference years: an inconsistent trend over the various months is highlighted.

In March 2020, a reduction in STEMI diagnoses was highlighted, the peak pandemic period, compared with March 2019 (OR 0.76 CI [0.60–0.93], *p* = 0.011). This difference disappeared in 2021 (OR 1.06 CI [0.90–1.34], *p* = 0.36).

The number of STEMI carried by 118 was higher during August 2020 than other years, (OR 1.28 CI [1.05–1.55], *p* = 0.01).

In Table 1, the average time to first EMS vehicle on the scene increased significantly, i.e., approximately 3 min, in 2020 and 2021 compared with 2019. In addition, the mean overall duration of the missions also increased significantly, i.e., approximately 7 min, in 2020 and 2021 compared with 2019. 

**Figure 2:**
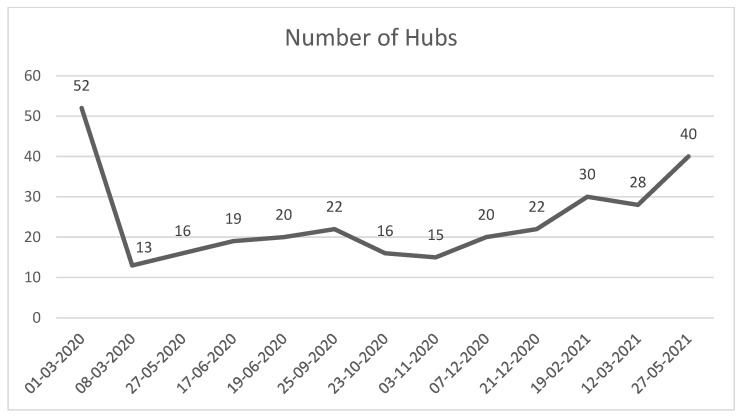
Number of hub hospitals active for STEMI in the Lombardy region from the start of outbreak.

The number of active HUB hospitals for STEMI patients in the Lombardy region significantly decreased from March 2020 (Figure 2). Figure 2 shows the number of active HUB hospitals for STEMI patients in the Lombardy region. After 8 March 2020, the regional health authority reduced the number of HUB centers. The number of HUBs for STEMI increased slowly during the first years of the pandemic, while after 27 May 2021, the number of HUBs returned to the same as the pre-pandemic era.

## 4. Comment

During the COVID-19 pandemic, a reduction in STEMI diagnoses, performed by EMS, was observed in the Lombardy Region, especially during the peak outbreak in March 2020 compared with March 2019, consistent with previous reports [11,25]. Contrariwise, with the conclusion of the pandemic period in March 2021, such a reduction in STEMI diagnoses was not registered anymore. In the three years covered by this study, there were no significant differences in the monthly average number of STEMI diagnoses. Conversely, during the months of the first pandemic wave, significant differences were highlighted, while in the following months, no diagnoses reduction was recorded, especially in the second wave (November and December 2020) and third wave (March and April 2021) of the COVID-19 pandemic. This last finding may be explained by the EMS reorganization and local authorities’ intervention, such as the increase in BMV vehicles and HUB hospitals.

Similarly to Marrazzo F., et al. [28], we observed a significant increase in the time taken for the first EMS vehicle on the scene and the overall rescue mission duration until May 2021.

Although the times increased, it is important to underline that this did not affect the proper implementation of guidelines that stipulate 120 min from the STEMI onset to balloon in the pre-hospital setting; perhaps a study of in-hospital times after the patient access in ED, could highlight a change in time PCI interventions, which could lead to a change in in-hospital clinical protocol, for a better understanding of the change in time of door-to-ballon. As depicted in Figure 2, we highlight the number of active HUB centers in the Lombardy region; the regional health authority closed several centres to guarantee new wards for COVID-19 patients. In Lombardy, 52 centres were available for advanced STEMI treatment in the pre-pandemic era, and during the pandemic peak, they were reduced to 13; this may be one reason explaining the rise in missions’ duration. This study shows how the pandemic changed transportation times. However, it was not necessary to set up therapeutic and assistance protocols other than those already present, in the pre-hospital setting. Furthermore, although the pandemic was better controlled during April and May 2021, the rescue times were still longer. The cause may be the dressing-time necessary for rescuers, who were obliged to wear all PPE to prevent COVID-19 transmission, and the vehicles’ sanitization times.

This study also reports that the number of STEMI diagnoses in August 2020 was higher than in both August 2019 and 2021. This finding could be explained by the reduction of holiday departures during the first year of the pandemic due to widespread apprehension and COVID-19 restriction policies.

Our study has several limitations. First, during the COVID-19 pandemic, AREU coordinated many rescue missions, and during the phases of greater workload, operators may have performed fewer ECGs in the pre-hospital, this phenomenon could be partially linked with the reduction of STEMI diagnoses made during the first wave. Secondly, we cannot exclude that the reduction of STEMI diagnoses in March 2020 may be linked to the higher number of cardiac arrests that occurred during the pandemic, as demonstrated by Baldi et al. [17]. Likewise, we are not able to ascertain whether the patients not intercepted by EMS may have decided to access the ED in a different manner. Further studies are necessary to clarify this issue. An important limitation is related to the diagnoses of STEMI either being performed by the EMS teams or by BMV crews through ECG execution and the subsequent analysis by SOREU staff; indeed, ECGs performed by BMV crews may be registered in patients displaying early symptoms with no chance to highlight an ST-elevation due to the quick intervention with the ECG being recorded before the infarction. Furthermore, some patients may access the ED spontaneously. These scenarios contribute to the reduction in the number of recorded STEMIs in the AREU registry.

## 5. Conclusions

During the 2020 and 2021 pandemic period, the Lombardy region did not present a significant reduction in STEMI diagnoses by the EMS system. Accordingly, the average time to first vehicle on the scene and the duration of missions increased in 2020 and 2021 compared to 2019, although this rise did not modify the pre-hospital clinical protocols, thanks to the fact that during the pandemic, the hospitalization of patients with STEMI took place according to the required standards.

## Figures and Tables

**Table 1:** Average time to first EMS vehicle on the scene and overall duration of the missions.

	Time to First Vehicle on the Scene (min)			Duration of the Missions (min)		
	2019	2020	2021	2019	2020	2021
**January**	13.6	13.3	15.5	61.0	58.6	66.0
**February**	12.0	12.9	14.3	55.1	55.5	62.5
**March**	12.1	17.8	15.4	56.3	73.9	64.4
**April**	12.1	15.0	15.1	59.0	68.8	66.1
**May**	12.8	14.8	15.0	58.3	64.8	61.7
**June**	12.5	14.5	13.7	56.2	62.4	58.9
**July**	12.7	15.2	14.8	54.3	61.2	60.8
**August**	13.2	14.6	14.0	57.2	64.1	62.2
**September**	12.4	13.9	14.1	57.4	59.8	63.4
**October**	12.5	15.9	13.8	59.0	63.2	60.4
**November**	12.7	15.7	14.0	59.9	74.6	62.5
**December**	13.3	15.8	15.6	59.0	68.9	67.0
	12.7 ± 0.3	15.0 ± 1.3 *	14.6 ± 0.7 *	57.7 ± 2.0	64.7 ± 5.9 *	63.1 ± 2.3 *

* *t*-test for paired data, *p* < 0.05.

## Data Availability

The data presented in this study are available on request from the corresponding author. The data are not publicly available in accordance with national data safety guidelines.
